# How, and why, science and health researchers read scientific (IMRAD) papers

**DOI:** 10.1371/journal.pone.0297034

**Published:** 2024-01-22

**Authors:** Frances Shiely, Kerrie Gallagher, Seán R. Millar

**Affiliations:** 1 Trials Research and Methodologies Unit, HRB Clinical Research Facility, University College Cork, Cork, Ireland; 2 School of Public Health, University College Cork, Cork, Ireland; West University of Timisoara: Universitatea de Vest din Timisoara, ROMANIA

## Abstract

**Objectives:**

The purpose of our study was to determine the order in which science and health researchers read scientific papers, their reasons for doing so and the perceived difficulty and perceived importance of each section.

**Study design and setting:**

An online survey open to science and health academics and researchers distributed via existing research networks, X (formerly Twitter), and LinkedIn.

**Results:**

Almost 90% of respondents self-declared to be experienced in reading research papers. 98.6% of the sample read the abstract first because it provides an overview of the paper and facilitates a decision on continuing to read on or not. Seventy-five percent perceived it to be the easiest to read and 62.4% perceived it to be very important (highest rank on a 5-point Likert scale). The majority of respondents did not read a paper in the IMRAD (Introduction, Methods, Results And Discussion) format. Perceived difficulty and perceived importance influenced reading order.

**Conclusion:**

Science and health researchers do not typically read scientific and health research papers in IMRAD format. The more important a respondent perceives a section to be, the more likely they are to read it. The easier a section is perceived, the more likely it will be read. We present recommendations to those teaching the skill of writing scientific papers and reports.

## Introduction

Reporting in the form of a peer-reviewed research paper, also known as a journal publication or research manuscript, is essential to the healthcare and science professions. The skill of writing a peer reviewed paper is highly specialized and challenging. It is also a challenge to teach this skill, yet it is essential to do so, as students are often required to engage with complex academic texts as well as write scientific reports [1–3]. Other cited reasons are: (i) increasing scientific literacy; (ii) staying informed of progress in a particular field of study; (iii) understanding the causation, clinical features, and natural history of a disease; (iv) evaluating the effectiveness of diagnostic tests and clinical therapies; and (v) determining whether there is support for or opposition to a particular argument [4–6]. Additionally, it is imperative that the reader is able to identify robustly designed research in order to make informed recommendations regarding policy or patient care [7].

Currently, most healthcare research papers are presented in the IMRAD format: **I**ntroduction (why the authors decided to do the research), **M**ethods (how they did it and how they chose to analyse their results), **R**esults (what they found), **A**nd **D**iscussion (what they believe the results to mean) with a preceding abstract. However, there is no evidence-based research determining the suitability of this approach. Nevertheless, it provides a means for scientific communities to organise and structure their work effectively [8]. Within the scientific community, the approach is based on the notion that having a clear structure and procedures can help scientists produce better quality work. In addition, it is thought to reduce the risk of mistakes and oversights and ensure compliance with best practices in research (10). We know that the amount of time students spend reading academic material is estimated to be between seven and fourteen hours per week, which represents an important component of the academic schedule [3, 9, 10]. Therefore, research on the suitability of the IMRAD approach is important.

Professor Trisha Greenhalgh, author of the seminal text “How to read a paper: the basics of evidence-based medicine and healthcare” suggests that if you are deciding whether a paper is worthy of study, you should do so based on the design of the methods section [11]. This is largely opinion based and is not predicated on clear evidence. Anecdotal evidence suggests people choose to read and examine research papers in different ways, but the literature is scant on the topic. One UK study attempts to address the strategies used by researchers and students when reading primary research [12]. The authors report that individuals at different career stages value different sections of scientific papers, with novice readers finding the methods and results sections to be particularly challenging to decipher [12]. Similarly, a study conducted in the US examined and compared how faculty members and students in an undergraduate science course engaged with a primary research article [13]. Faculty and students were able to demonstrate understanding of the research design at some point during the reading process, however, the faculty displayed this ability almost four times as often as students [13]. Both of these studies are limited in their capacity and generalisability as they are restricted to students and researchers in the biological sciences.

From a teaching and learning perspective, we are interested in knowing more about how science and health researchers read IMRAD research papers and the importance they place on each section. Our primary aim is to establish the order in which these researchers read an IMRAD formatted paper and why. By establishing this, educators can better craft their teaching to ensure that students understand the importance of each section, have the knowledge and skills necessary to write an effective scientific paper or report, and the ability to critically appraise the work of others.

## Materials and methods

The survey (S1 File) was created on Google Forms by two members of the research team (KG and FS) and independently reviewed by two reviewers (ST and EM). The survey had three parts. Part 1 was concerned with written informed consent. When participants clicked the link, they were brought the informed consent page which provided details of the study, what was required from them, knowledge of the voluntary nature of the participation and right to withdraw at any stage, and the contact details of the principal investigator. To proceed, participants had to select either I consent to participate, which brought the participants to Part 2 of the survey, or I do not consent to participate, which meant the participants exited the survey. Part 2 of the survey collected data concerning the demographic characteristics of the respondents. Part 3 focused on questions pertaining to the order in which researchers read a primary research paper, how easy it is to read each of the sections (based on a 7-point scale) and how important each section of a primary research paper is for its understanding. The survey also assessed when a reader stops reading and why. The style of questions was mixed and included Likert scale ratings and closed and open-ended questions. Ethical approval was granted by the Social Research Ethics Committee (SREC), University College Cork (Log 2021–165). Participants provided written informed consent.

### Recruitment

This was an online survey and recruitment was online. Inclusion criteria were: academics, health professionals, and patients and members of the public, involved in science or health research and/or teaching. Exclusion criterion was: under 18 years of age. Our recruitment strategy was to target academics, researchers and patient and public involvement members of our existing networks, all who work within or are affiliated with Universities in the UK, Ireland and Canada. The lead author, FS, is primarily associated with clinical trial networks. An email of invitation outlining the aim of the study and survey link was sent electronically via the Health Research Trial Methodology Research Network (HRB TMRN), Ireland (~3000 subscribers), Medial Research Council-National Institutes of Health and Care Research-Trial Methodology Research Partnership (MRC-NIHR-TMRP), UK, locally at University, University College Cork (all academic and research staff—~2500 people), via X, formerly known as Twitter (@FrancesShiely; @hrbtmrn) which was forwarded and liked and via LinkedIn (FS account). FS also distributed the link to her academic research partners in Ireland, UK, Hungary, Czech Republic, France, and Canada and asked them to forward to their respective Universities and contacts.

### Statistical analysis

We obtained 152 responses to the survey, 139 of which completed answers to the order in which they read the research paper. These were included in the analyses. Descriptive characteristics were examined for the full sample. Likert scale answers to reading order, perceived difficulty and perceived importance questions for each research paper section are shown as percentages. Reading order, perceived difficulty and importance ranking were also examined according to career stage. Observations were independent, with no individuals belonging to more than one career stage group. Relationships between perceived difficulty ranking, perceived importance ranking and research paper reading order were also examined using Spearman’s rank-order correlation. Data analysis was conducted using Stata SE Version 13 (Stata Corporation, College Station, TX, USA) for Windows. For all analyses, a p value (two-tailed) of less than .05 was considered to indicate statistical significance. Qualitative variables were summarised according to the most frequent occurrence to provide a picture on the reasons participants chose to read a paper in their chosen format.

## Results

Table 1 shows descriptive characteristics of the study respondents. The majority of subjects were female (61.2%), 90.7% were under 60 years of age and 94.7% reside in Europe. Study respondents included MSc and PhD students (n = 17), early-career researchers (n = 39), mid-career researchers (n = 36) and established/leading researchers and research managers (n = 39). A majority (88.5%) worked in academic research at a university or college, with 61.9% indicating both research and teaching responsibilities. Almost 90% of respondents stated that they were experienced in reading a research paper.

**Table 1:** Characteristics of study respondents (n = 139).

Gender	Number and %
Male	46 (33.1)
Female	85 (61.2)
Non-binary	3 (2.2)
Prefer not to answer	5 (3.6)
**Age**	
20–29 years	11 (7.9)
30–39 years	51 (36.7)
40–49 years	35 (25.2)
50–59 years	29 (20.9)
60–69 years	11 (7.9)
Prefer not to answer	2 (1.4)
**Location**	
Europe	133 (95.7)
Asia	4 (2.9)
Australia	1 (0.7)
Other	1 (0.7)
**Career stage**	
MSc by research or PhD	17 (12.2)
Early-career researcher	39 (28.1)
Mid-career researcher	36 (25.9)
Established/Leading researcher or Research Manager	39 (28.1)
Other	8 (5.8)
**Place of work**	
University/College–Academic or researcher	123 (88.5)
University/College–Administration	7 (5.0)
Non-Profit Organisation	3 (2.2)
Private research company	1 (0.7)
Other	5 (3.6)
**Work description**	
Research and teaching	86 (61.9)
Research only	38 (27.3)
Research administration only	6 (4.3)
Research management only	8 (5.8)
Other	1 (0.7)
**Experience in reading a research paper**	
Yes (experienced)	125 (89.9)
No (not experienced)	14 (10.1)

### Reading order, perceived difficulty and perceived importance for IRMAD sections

**Fig 1:**
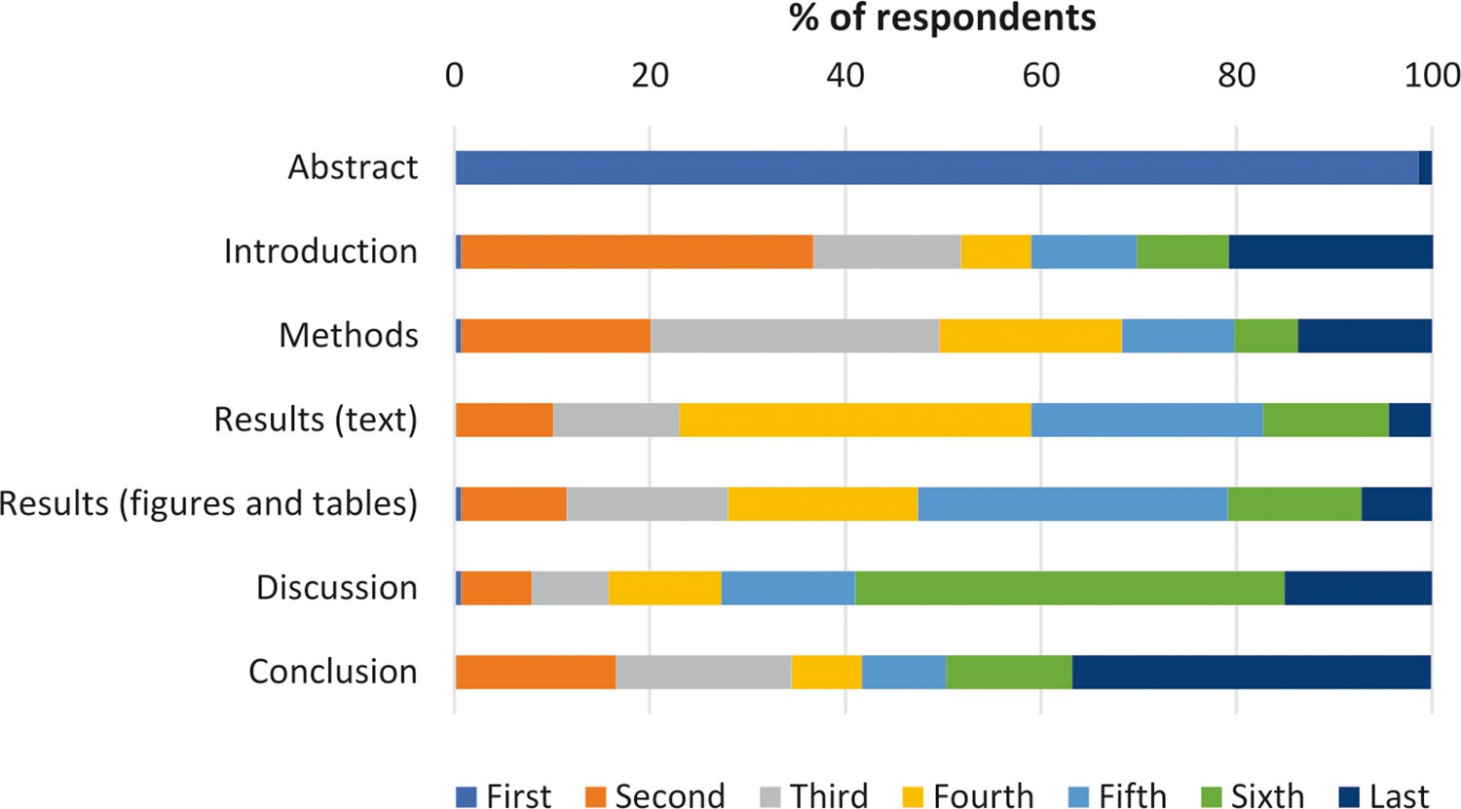
Research paper reading order, for each section, according to study respondents (n = 139).

Fig 1 shows research paper reading order according to each section. A majority of respondents (98.6%) indicated that they read the abstract section first when reading a scientific paper, with 36.0% (Introduction), 29.5% (Methods), 36.0% (Results–text), 31.7% (Results–figures & tables), 43.9% (Discussion) and 36.7% (Conclusion) of subjects stating that they read these sections second, third, fourth, fifth, sixth, and last, respectively. Noticeably, a majority of respondents indicated that they did not read a paper in the IMRAD order; for instance, while over one-third of respondents stated that they read the Introduction section second, 64.0% did not, with just over one-fifth (20.9%) indicating that they read this section last.

We asked respondents why they read in their preferred order. For the 98.6% (149/152) who selected the abstract first, the reasons can be summarised as the fact the abstract gives an overview or summary of the paper and it allows one to see if the paper is worth continuing reading (“the abstract gives the summary and informs as to whether I will read the whole paper”, “abstract gives a feel for quality of the paper”, “fast and easy”, “the abstract has the main points and is usually freely available”). We were interested in what respondents read after the abstract. For those who read the introduction second, i.e., the IMRAD format (only two read the introduction first), the dominant reason was because it’s logical to read in the order it’s presented (“it’s the logical order”, “I usually read papers in the order it is written”). For those who chose the methods second, 21.2% (31/146) the reasons can be themed as to ensure robustness or quality of the study (“methods to understand whether it was well conducted”, “checking the methods to ensure it is relevant to me”, “understand how the methods led to such results”, “is this something I can trust”). Only 15 people read the results section second, regardless of whether it was the results-text or results-figures & tables. The key reasons for reading the results second can be summarised as establishing the findings (“get right to the results”, “results to understand the main findings”, “the results are arguably the most important part of the document”, “do the results show what they are saying?”). Those choosing to read the discussion second, 8.1% (12/149), did so to establish the key findings (“the discussion and conclusion is the essence of what the study found”, “discussion and conclusion are most interesting”, “discussion to see if anything interesting came out of the results”). For those who read the conclusion second, 17% (25/146), the reasons are summarised as establishing the overall view of the paper and if the research is of value “see if a paper of value”, “know if it’s useful to me”, “final result/outcome”, “overall view”, “clarifies what the author perceives to have been achieved”, “I read the conclusions to build on the summary conclusions of the abstract”.

**Fig 2:**
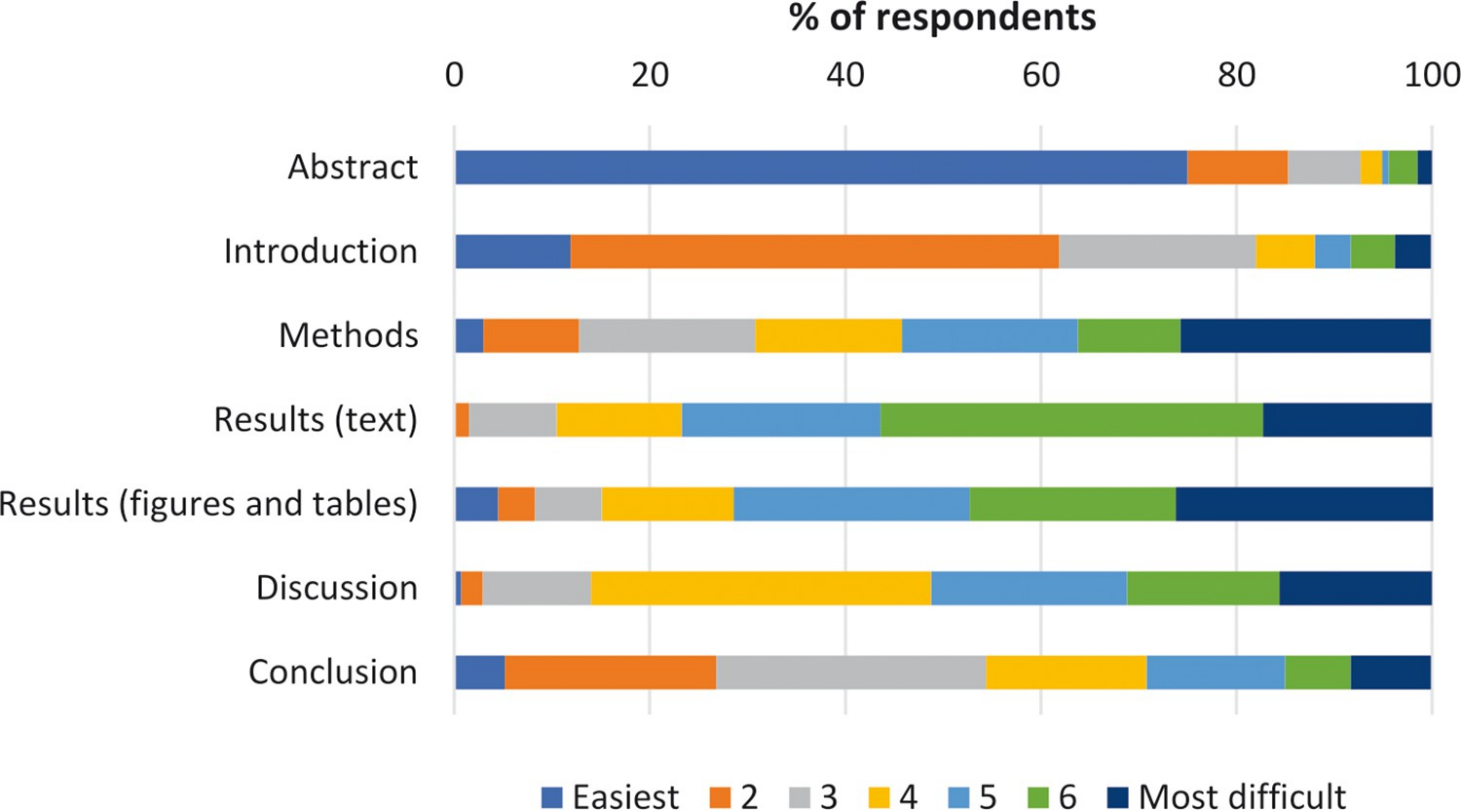
Perceived difficulty when reading a research paper, for each section, according to study respondents (n = 139).

To explore perceived difficulty when reading a research paper, participants were asked to rank a series of questions according to reading difficulty on a 7-point scale, 1 being the easiest and 7 being the most difficult (Fig 2). Similar to reading order, a majority (75.0%) of respondents stated that they found the Abstract section to be easiest to read (rank 1). The Introduction and Conclusion sections were perceived as next easiest, respectively. Taking the blue and orange together (ranks 1 and 2) the same trend applies. On the opposite end of the scale (rank 7-most difficult, the dark navy colour), the Results-figures & tables section, was perceived to be most difficult (26.3%), followed by the Methods (25.6%) and Results-text (17.3%).

**Fig 3:**
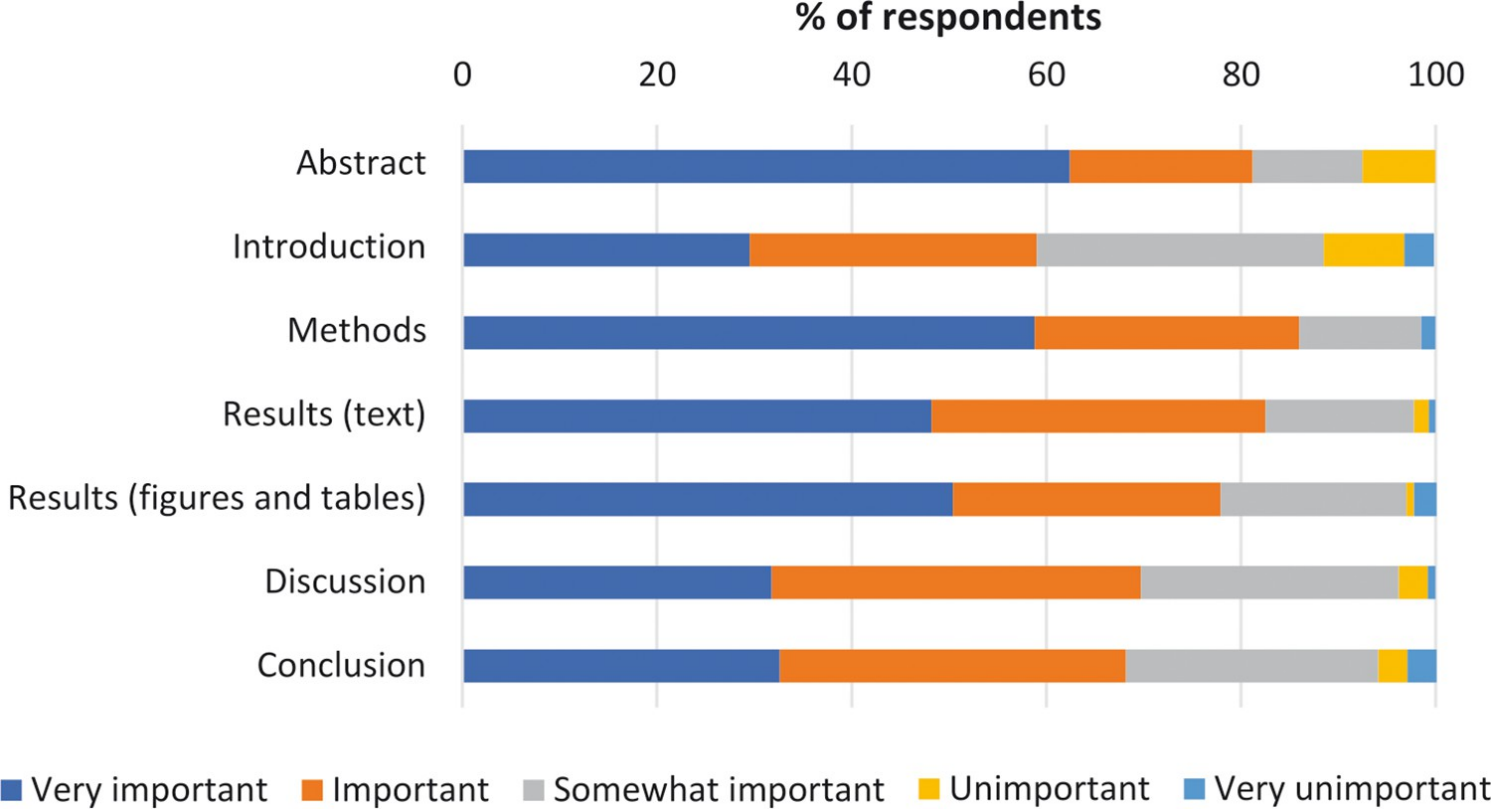
Perceived importance when reading a research paper, for each section, according to study respondents (n = 139).

Perceived importance for each section was assessed using a 5-point Likert scale (Fig 3). The Abstract and Methods sections were perceived as very important by 62.4% and 58.8% of respondents, respectively. Although few respondents perceived any section as unimportant or very unimportant, only 29.5%, 31.8% and 32.6% of subjects believed that the Introduction, Discussion and Conclusion sections were very important.

### Reading order and career stage

**Fig 4:**
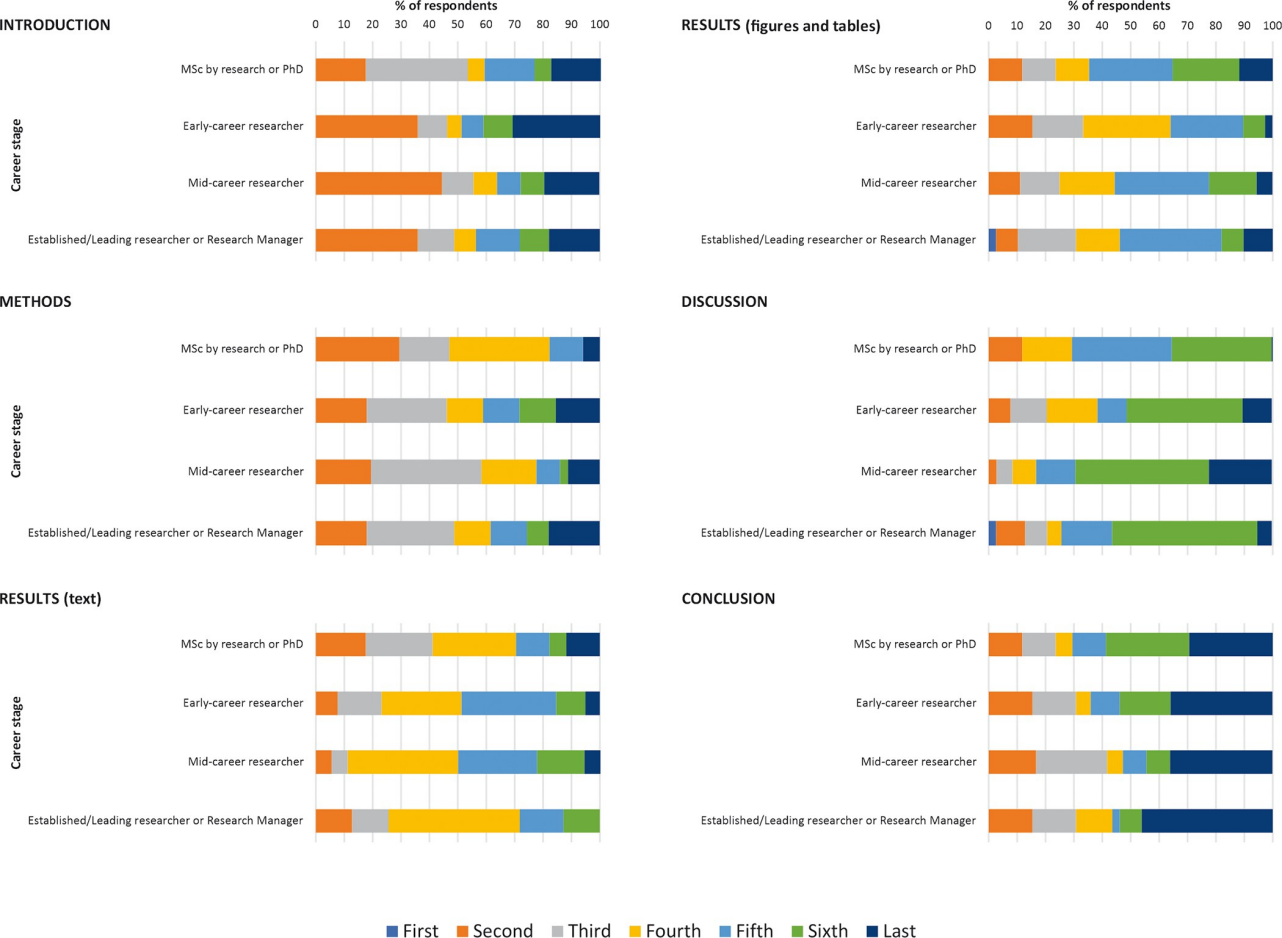
Research paper reading order, for each section, according to career stage.

Fig 4 shows the different sections of the research paper in reading order according to career stage. Differences in reading order were noted, with the greatest differences in reading order observed in the Results-text, Results-figures & tables and Discussion sections. Notably, 46.2% of established/leading researchers or research managers read the Results-text section fourth (in IMRAD order), compared to 29.4% of MSc by research/PhD students and 28.2% of mid-career researchers who did so. Similarly, a higher percentage of established/leading researchers or research managers indicated reading the Results-figures & tables and Discussion sections according to IMRAD reading order when compared to other career stages.

### Perceived difficulty and importance for each section according to career stage

**Fig 5:**
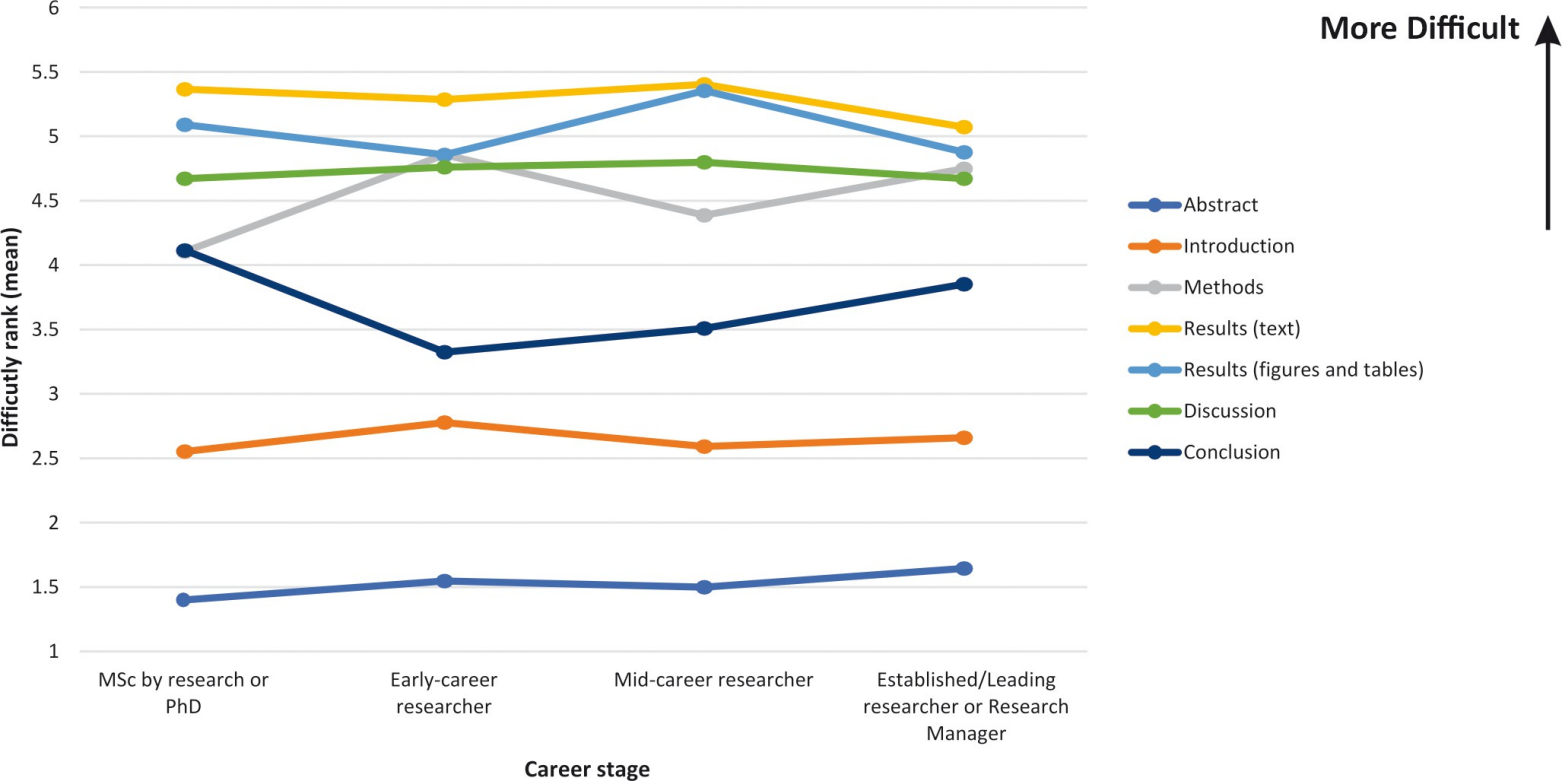
Perceived difficulty when reading a research paper, for each section, according to career stage.

**Fig 6:**
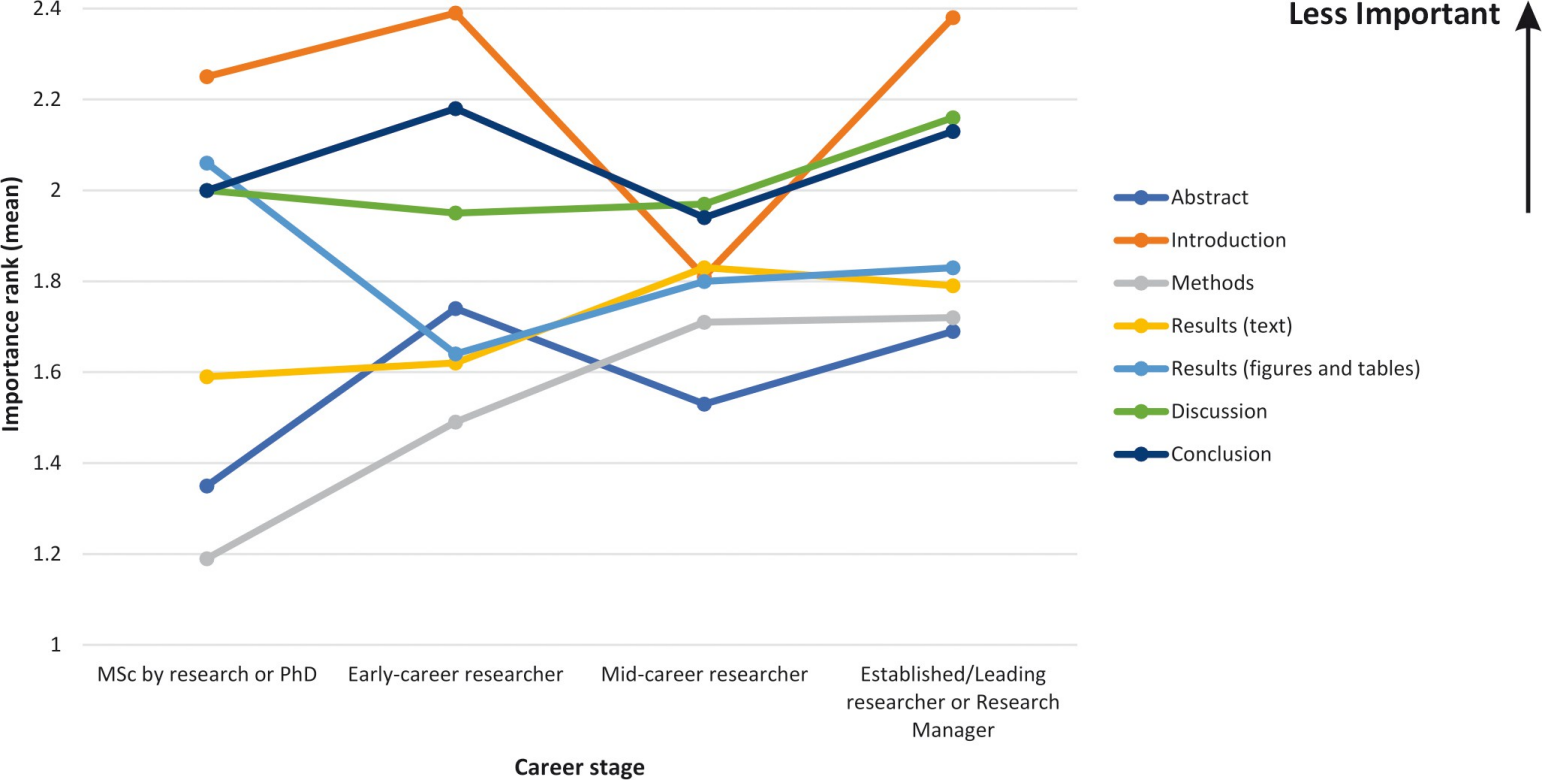
Perceived importance when reading a research paper, for each section, according to career stage.

Perceived difficulty ranking and perceived importance ranking according to career stage, for each IMRAD section and the abstract, are shown in Figs 5 and 6. Consistent with results observed among all subjects, regardless of career stage, the Results-text and Results-figures & tables sections and Discussion sections were perceived as most difficult to read. Differences were found to be greatest for the Conclusion section, with MSc by research or PhD students being more likely to rank this section as difficult to read. With regard to the Introduction section, mid-career researchers were more likely to rank this section as important. Interestingly, MSc by research/PhD students were more likely to rank the Methods section as being important when compared to other career stages.

### Correlations between perceived difficulty, importance and reading order

Spearman correlation coefficients between the ranking of perceived difficulty, perceived importance and reading order, according to each section, are shown in Table 2. Significant correlations between perceived difficulty ranking and reading order were observed for the Methods (rho = 0.450, p < .001), Results-figures & tables (rho = 0.333, p < .001), Discussion (rho = 0.204, p = .018) and Conclusion (rho = 0.334, p < .001) sections, indicating that the easier a respondent perceived that section to read, the more likely they were to read it at an earlier stage. Significant correlations between perceived importance ranking and reading order were observed for the Introduction (rho = 0.467, p < .001), Methods (rho = 0.426, p < .001), Results (text) (rho = 0.250, p = .003), Results (figures & tables) (rho = 0.173, p = .048), Discussion (rho = 0.214, p = 0.14) and Conclusion (rho = 0.302, p < .001) sections, suggesting that the more important a respondent perceived that section to be, the more likely they were to read it at an earlier stage.

**Table 2:** Spearman correlation coefficients between perceived difficulty ranking, perceived importance ranking and research paper reading order, according to each section.

	Abstract		Introduction		Methods		Results (text)		Results (figures & tables)		Discussion		Conclusion	
	rho	p	rho	p	rho	p	rho	p	rho	p	rho	p	rho	p
**Perceived difficulty**	0.168	.051	0.136	.117	0.450	**< .001**	0.169	.051	0.333	**< .001**	0.204	**.018**	0.334	**< .001**
**Perceived importance**	0.168	.053	0.467	**< .001**	0.426	**< .001**	0.250	**.003**	0.173	**.048**	0.214	**.014**	0.302	**< .001**

Values are presented as Spearman correlation coefficients (rho) between perceived difficulty ranking, perceived importance ranking and research paper reading order, for each section (n = 139).

Significant p **highlighted**.

### When and why respondents stop reading a paper

We asked respondents why they stopped reading a research paper. The main sections were (in no particular order) results, introduction, methods and abstract. Of the 98.6% that read the abstract first, 28% (42/149) then stopped reading at this stage. The reason given in all cases is lack of relevance (“not relevant to my interests”, “will have identified if it is of relevance”). The main reasons for those that stop reading at the introduction is the writing style (“poorly written”, if the writing style is overly complex”, it’s too dense and not interesting”, not relevant, poorly conducted”. For those who stop reading at the results section, the main reasons given are the results are too complex or poorly explained (“it gets too difficult to understand”, “paper is not relevant”, “too complicated”, “it’s no longer relevant if results are not clear”). For those who stop reading at the methods section, the main reason is they are unclear or too difficult or there is a perception that the methods are not needed (“becomes technical and I don’t’ need more details”, the methods might not be interesting to what I am trying to learn from reading the paper”, generally for my work methods are not important”, not interested in it, I Know already by the methods if I ‘like’ the paper”, “most often the methodology is not clear enough”).

## Discussion

We know most research papers are published in IMRAD format, preceded by an abstract. We sought to establish if researchers, and at different career stages, typically read a paper in this way. We found that even though most researchers consider themselves experienced readers of primary research papers, respondents did not typically engage with the literature in IMRAD format. Reading strategies varied depending on perceived difficulty and perceived importance of the paper sections. The more important a respondent perceived the section to be, the more likely they were to read it at an earlier stage. Almost all science and health researchers read the abstract first, and a significant proportion stop reading there. The primary reason for stopping is lack of relevance.

We can see very clearly that the abstract is read first by most researchers, regardless of career stage, perceived to be the most important, and also perceived to be the easiest to read. While there isn’t prior research to compare this finding to, we surmise that it is because it is a summary of the overall paper, and a logical place to begin. It’s also possible that it is because it is presented first in all research papers, or for pay per view journals/papers it is usually available when the rest of a paper is beyond a paywall. It could be that researchers know the abstract is used by journal editors to decide if it is worth continuing to read a paper to decide if it should be peer-reviewed or it could be that the abstract is often used in systematic reviews when screening (typically title and abstract) and thus habitually researchers read it first. However, we don’t know any of this for sure and would need a qualitative study to confirm or refute these. We do know the reasons that a significant number of people stop reading the abstract and that is relevance, or the lack of it. Another consideration might be the reading level at which the abstract is pitched. We have conducted a previous study on the readability of trial lay summaries, which are written for a lay audience [14]. We found that no lay summary met the recommended reading age for health care information of 11–12 years. None of them were considered "easy" to read, in fact over 85% were considered "difficult" to read [14]. By extension we can assume the scientific abstract, which we have considered here, will be no better, and likely worse, and thus challenging for a novice researcher. We recommend that when teaching the skill of writing a scientific paper, or report, in the science or health research disciplines, teachers emphasise the importance of the abstract and give consideration to the target audience when formulating their approach. Online readability tools are readily available to assist the process, but we do not recommend relying on them solely [14].

Further evidence of the complexity of reading different parts of scientific papers is the response to the methods section. The evidence that does exist on how to read a paper suggests that if you are deciding whether a paper is worthy of study, you should do so based on the design of the methods section. As mentioned previously, this was opinion based rather than evidence based [3]. Our respondents typically considered the methods section to be of low importance on the 7-point Likert Scale and some stopped reading there due to the unclear language or technical nature of the section. This result was unexpected, as there is a general consensus in the scientific community that the methods section is considered one of the most important sections of any research paper, given that it provides essential insight into the conduct of the study and its integrity, the conclusions derived from them, and the reproducibility of the work [3]. Indeed, across several well recognized and validated critical appraisal tools, including the Critical Appraisal Skills Program (CASP) for Randomized Control Trials [15], the ROB 2.0 Risk of Bias Tool [16] and the ROBINS-I Risk of Bias for non-randomized (observational) studies [17], focus is directed towards systematically examining the methods section in order for the reader to determine the strength of the evidence presented, its reliability and relevance to clinical practice.

### Strengths and weaknesses

We had a reasonable response (n = 139) but we are unable to calculate a response rate due to the mode of distribution, a significant weakness to the study. Our study likely demonstrates selection bias, given the known research networks through which the survey was distributed are all funded through academic grants and the majority of our respondents were academic researchers working in a University/College. The research would be enhanced if we had a larger response from non-profit organisations. There were only six respondents outside of Europe and this is a weakness in terms of generalisability. However, on the positive side, non-response bias was not evident, and we had a full dataset for 139 respondents.

### Recommendations

The lessons for future practice are:

- Ensure your abstract gives enough detail to ensure relevance and pique interest because if you don’t, science and health researchers will lose interest and stop reading;
- Ensure the introduction is well written because if it is poorly written, you will lose the reader (we suggest using the freely available online readability scales, e.g., Flesch Reading Ease Score (FRES), Flesch-Kincaid Grade Level (FKGL), Simplified Measure of Gobbledegook (SMOG), Gunning Fog (GF), Coleman-Liau Index (CLI), and Automated Readability Index (ARI) readability scales and paying attention to Plain Language guidelines [18], e.g., in the UK the Plain English UK guidelines are most relevant;
- Don’t make the methods section too technical. Find the balance between overcomplicating the methods and giving enough detail so the study can be replicated;
- Keep the results simple and explain them well.

## Conclusions

This study provides an insight into the order in which IMRAD papers are read and the reasons for doing so. Existing evidence says that to determine if a paper is worthy of reading, you should read the methods section to decide. Our results refute this and show the methods section to be one of the sections perceived most difficult to read and also the least important. Our results show the importance of the abstract to the scientific and health research community, and we recommend when teaching the skill of scientific writing a particular focus is given to the abstract. Future research on this topic is welcome in a more diverse and larger sample.

## Supporting information

S1 File Undergraduate student survey. (PDF)
